# Supplementary material for: Causal Relationship between Aging and Anorexia Nervosa: A White-Matter-Microstructure-Mediated Mendelian Randomization Analysis
Source: Biomedicines. 2024 Aug 16;12(8):1874. doi: 10.3390/biomedicines12081874 (PMC11351342; doi:10.3390/biomedicines12081874)
Supplement: Supplementary file 1 [file biomedicines-12-01874-s001.zip › Supplementary Figure/Figure S8/Leaveoneout plot of MR analysis for TL to ubm-b-1883.pdf]

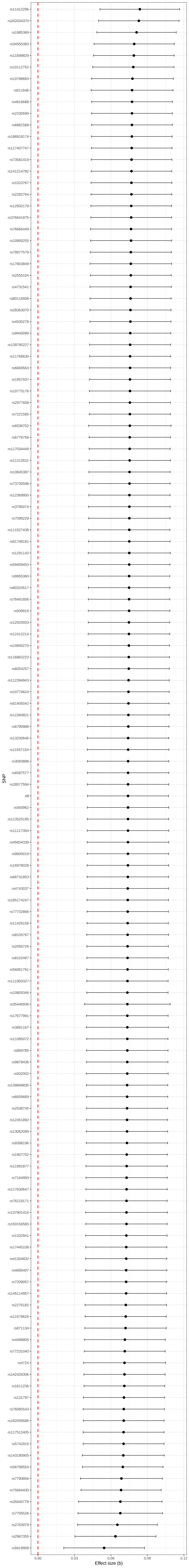

rs11412296
rs202034370
rs1985369
rs34550383
rs11699829
rs10112752
rs10768683
rs611646
rs4616688
rs2230590
rs6881568
rs188918174
rs117407747
rs73581419
rs141214782
rs1023767
rs2282764
rs12932179
rs376641875
rs76666449
rs10905255
rs79977579
rs17803849
rs2555104
rs4731541
rs80116508
rs28363070
rs4530278
rs9940099
rs139795227
rs11769630
rs6669563
rs1957937
rs10773176
rs2977608
rs7221585
rs6536702
rs6776756
rs117034449
rs11212631
rs10845387
rs73730598
rs12369950
rs3785074
rs7099229
rs111527438
rs61748181
rs1291143
rs59409453
rs9955360
rs80324517
rs78491606
rs939916
rs12925933
rs12412214
rs10840270
rs116863223
rs6054257
rs112394943
rs10774624
rs61405042
rs11584821
rs6790988
rs13230646
rs11557154
rs3093888
rs6587577
rs28577594
All
rs450962
rs113525195
rs11117354
rs45604339
rs9600019
rs16978028
rs66731853
rs4743037
rs185174247
rs77732866
rs11426156
rs8105767
rs2056726
rs8102497
rs56061761
rs111950327
rs10805346
rs35446936
rs17677991
rs3891167
rs11085072
rs869785
rs9878436
rs932002
rs139669835
rs6659669
rs2538745
rs12451892
rs13062095
rs9398196
rs1907702
rs11991877
rs7164950
rs117630647
rs76219171
rs137901416
rs150150565
rs1332941
rs17445108
rs41304832
rs4695407
rs7209057
rs145114957
rs2276182
rs11579626
rs871134
rs4498805
rs77231040
rs4724
rs142426306
rs1611236
rs131797
rs76065543
rs182059586
rs117512405
rs5742915
rs143190905
rs56799554
rs7790856
rs75664430
rs35640778
rs7705526
rs2763979
rs2967355
rs9419958
SNP
0.00
0.03
0.06
0.09
0.12
Effect size (b)
